# The Serine 814 of TRPC6 Is Phosphorylated under Unstimulated Conditions

**DOI:** 10.1371/journal.pone.0018121

**Published:** 2011-03-23

**Authors:** Simon M. Bousquet, Michael Monet, Guylain Boulay

**Affiliations:** Department of Pharmacology, Université de Sherbrooke, Sherbrooke, Québec, Canada; University of Oldenburg, Germany

## Abstract

TRPC are nonselective cation channels involved in calcium entry. Their regulation by phosphorylation has been shown to modulate their routing and activity. TRPC6 activity increases following phosphorylation by Fyn, and is inhibited by protein kinase G and protein kinase C. A previous study by our group showed that TRPC6 is phosphorylated under unstimulated conditions in a human embryonic kidney cells line (HEK293). To investigate the mechanism responsible for this phosphorylation, we used a MS/MS approach combined with metabolic labeling and showed that the serine at position 814 is phosphorylated in unstimulated cells. The mutation of Ser^814^ into Ala decreased basal phosphorylation but did not modify TRPC6 activity. Even though Ser^814^ is within a consensus site for casein kinase II (CK2), we showed that CK2 is not involved in the phosphorylation of TRPC6 and does not modify its activity. In summary, we identified a new basal phosphorylation site (Ser^814^) on TRPC6 and showed that CK2 is not responsible for the phosphorylation of this site.

## Introduction

Calcium homeostasis is crucial for every cell type, and its tight regulation allows intracellular calcium to be a widely used second messenger [1]. This regulation is a fine balance between calcium entry from the extracellular medium, release from intracellular stores, and extrusion through the activity of pumps or exchangers. TRP (transient receptor potentials) are plasma membrane-embedded calcium channels that were first discovered in *Drosophila melanogaster.* Up to 28 mammalian isoforms of dTRP have been cloned so far and have been distributed into six subfamilies (TRPC, TRPV, TRPM, TRPP, TRPML, and TRPA) [2]. TRPCs are the closest subfamily to dTRP and include seven members (TRPC1 to TRPC7). Their role as calcium channels has been well characterized, but their exact activation and regulation mechanisms have yet to be fully understood. Along with STIM and Orai, TRPCs are involved in store-operated and receptor-operated calcium entry following hormonal stimulation of Gq-protein coupled receptor or receptor tyrosine kinase [3]. These receptors activate phospholipase Cβ or phospholipase Cγ, which hydrolyze phosphatidylinositol-4,5-bisphosphate into diacylglycerol and inositol 1,4,5-trisphosphate. inositol 1,4,5-trisphosphate activates its receptor on the endoplasmic reticulum to induce calcium release. Store depletion and diacylglycerol formation activate channels located at the plasma membrane to induce Ca^2+^ entry as long as the stimulation is maintained. TRPC6 is one of these Ca^2+^ entry channels [4]. A dysregulation of TRPC6 has been associated with idiopathic pulmonary arterial hypertension [5], [6], focal segmental glomerulosclerosis [7], [8], and hyperproliferation of cancer cells [9]. Understanding the mechanisms regulating TRPC6 activity and routing is thus essential to better treat or prevent these pathologies.

Post-translational modification of TRPCs has been shown to influence their activity and routing. Following EGFR activation, TRPC6 is phosphorylated by Fyn, a Src family protein tyrosine kinase. Phosphorylation by Fyn allows optimal activation of TRPC6 [10]. Protein kinase G phosphorylates TRPC6 on Thr^69^, thus decreasing its channel activity [11]. Protein kinase C phosphorylates TRPC6 on Ser^768^ [12] and Ser^448^ [13]. We have shown that protein kinase C-dependent phosphorylation of TRPC6 on Ser^448^ decreases its activity and that TRPC6 is phosphorylated under basal conditions [13]. The purpose of the present study was to investigate the mechanism responsible for this basal phosphorylation of TRPC6. Using a mass spectrometry approach, we found out that the Ser^814^ was phosphorylated and contributed to 50% of the basal phosphorylation state of TRPC6. Surprisingly, mutant TRPC6^S814A^ displayed an activity similar to that of wild-type TRPC6. As Ser^814^ is within a consensus sequence for phosphorylation by CK2, we used two CK2 inhibitors to investigate the possible involvement of this kinase. However, the inhibition of CK2 did not modify the phosphorylation state or activity of TRPC6. Thus, we have identified a new phosphorylation site on TRPC6.

## Results

**Figure 1:**
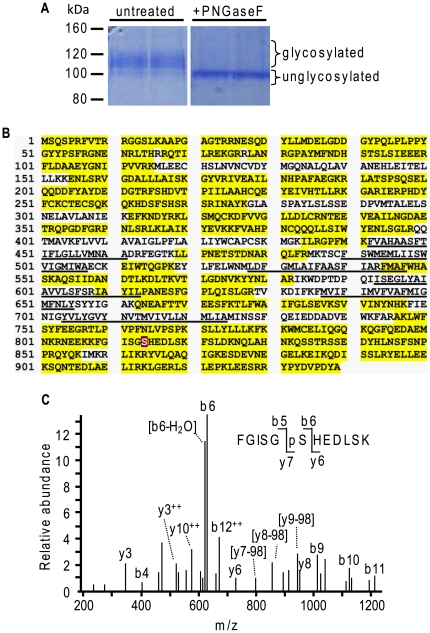
MS/MS identification of potential phosphorylated residues on TRPC6. **A**, Untreated T6.11 cells were lysed before TRPC6 was immunoprecipitated using an anti-HA antibody. The immunoprecipitated proteins were then deglycosylated with PNGaseF or not, before being separated by SDS-PAGE and stained with Colloidal Brilliant Blue. **B**, Sequence coverage of TRPC6 by nano-LC-MS/MS after tryptic digestion is highlighted. Ser^814^ is shown in white on a black background. The transmembrane segments are underlined. Residues 931 to 939 represent the HA epitope. Total sequence coverage is 68%. **C**, Simplified LC-MS/MS spectrum of the FGISGpSHEDLSK peptide from TRPC6 phosphorylated at Ser^814^. The phosphorylation of Ser^814^ was confirmed by the mass assignments of fragmentation ions b5, b6, y6, and y7.

HEK293 cells stably expressing TRPC6 (HEK293 T6.11) were solubilized, and TRPC6 was immunoprecipitated using an anti-hemagglutinin (HA) antibody and size fractionated by SDS-PAGE. The gel was stained with colloidal Brilliant Blue, and the bands corresponding to TRPC6 (Fig. 1A) were excised and in-gel digested with trypsin. Tryptic fragments were analyzed by LC-MS/MS and identified by database searches using the Mascot search engine. Sequence coverage of TRPC6 totaled 68.1% (77.8% of intracellular regions) (n  =  2) (Fig. 1B). Nano-LC-MS/MS analyses and MS spectra revealed that Ser^814^, located in the C-terminus, was phosphorylated with an Ascore of 57.75. An Ascore of 20 or more means that the phosphorylation is on that particular residue with a probability of 99%, and not on another serine, threonine, or tyrosine that may be present on the peptide [18]. Figure 1C shows the tandem mass spectrometry (MS/MS) spectrum of phosho-Ser^814^.

**Figure 2:**
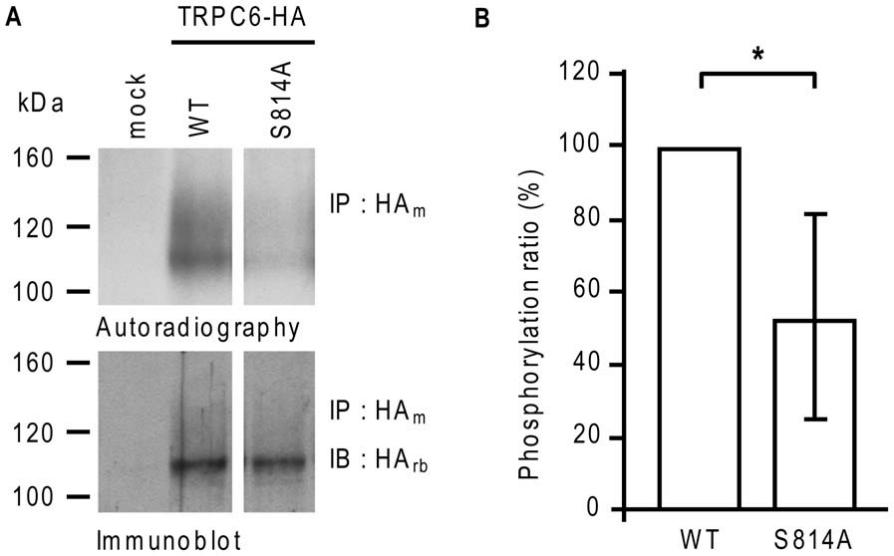
Metabolic labeling of TRPC6 and mutants reveals that Ser814 is phosphorylated. **A**, Metabolic labeling was carried out as described in *Experimental Procedures*. HEK 293T cells transfected with wild-type or mutant TRPC6 were lysed and TRPC6 was immunoprecipitated using a mouse monoclonal anti-HA antibody (HAm) and separated by SDS-PAGE. The autoradiogram (upper panel) and the immunoblot using a rabbit polyclonal anti-HA antibody (HAr) (lower panel) are representative of five independent experiments. **B**, The histogram represents the relative amount of phosphorylated TRPC6 calculated as the ratio of phospho-TRPC6 (densitometric analysis of autoradiograms) to total TRPC6 (determined by scanning and quantifying the immunoblot signals) for each sample. The histogram is the average ± SD of five experiments. * P<0.03.

To confirm that Ser^814^ was phosphorylated under unstimulated conditions, we substituted it for an alanine. HEK293 cells were transiently transfected with the HA-tagged TRPC6 or TRPC6^S814A^ mutant and metabolically labeled with inorganic ^32^P. HA-tagged proteins were then immunoprecipitated with an anti-HA antibody and size fractionated by SDS-PAGE. The phosphorylation states of the proteins were revealed by autoradiography. Under basal conditions, the level of phosphorylation of mutant TRPC6^S814A^ corresponded to 52.8±28.0% of that of WT TRPC6 (Fig. 2A and 2B). Mutation did not significantly alter the overall expression of TRPC6 (Fig. 2A, lower panel). Thus, as the substitution of Ser^814^ for Ala decreased the basal phosphorylation of TRPC6 by only 50%, these results suggested that Ser^814^ is a major phosphorylation site under unstimulated conditions. These results further suggest the existence of another unknown phosphorylation site on TRPC6.

**Figure 3:**
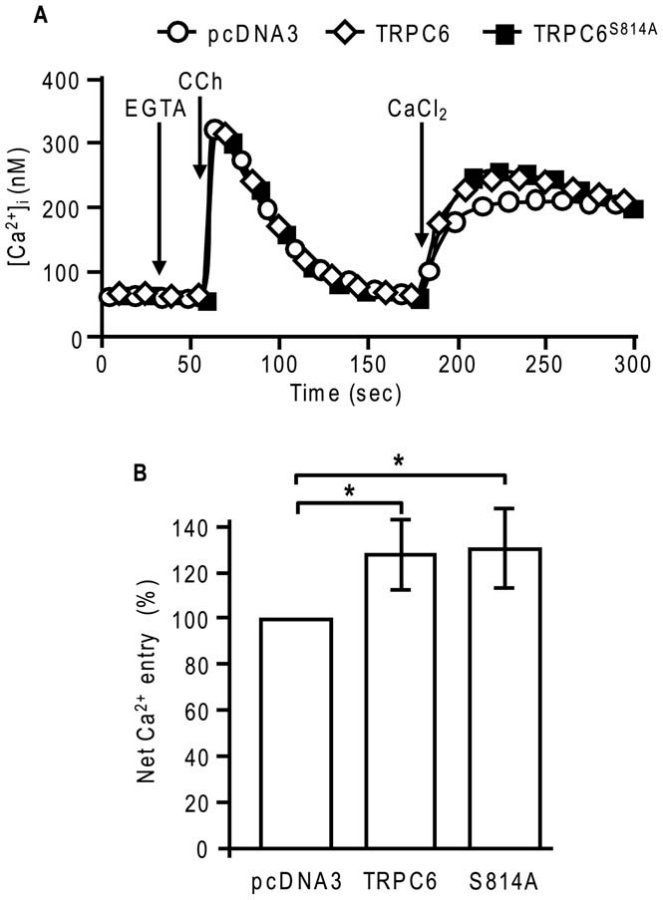
The S814A mutation does not alter TRPC6 activity. **A**, [Ca^2+^]_i_ was recorded in HEK293T transiently transfected with pcDNA3 (open circles), TRPC6WT (open diamonds), or TRPC6^S814A^ (closed squares). The cells were incubated in the absence of extracellular Ca^2+^ (in the presence of 0.5 mM EGTA) for 30 s before being stimulated with 5 µM CCh. External Ca^2+^ (1.8 mM) was restored at 180 s. The graphs represent the average of 103 to 165 cells from one representative experiment. **B**, CCh-induced net Ca^2+^ entry (average of [Ca^2+^]_i_ values after 174–177 s subtracted from [Ca^2+^]_i_ values after 222–228 s) were calculated and graphed as averages ± SD of six independent experiments. * *P<0.01*.

**Figure 4:**
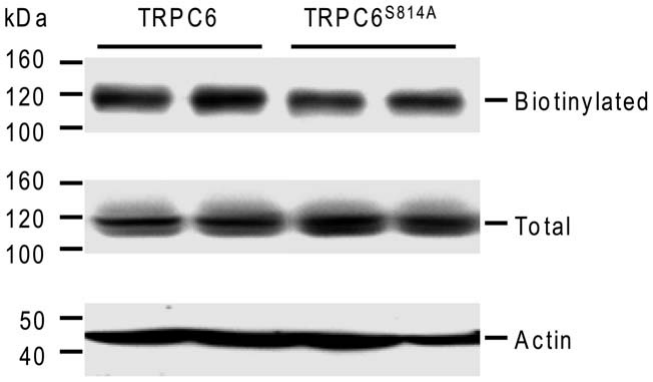
The mutation of Ser^814^ into Ala does not modify the level of expression nor the trafficking of TRPC6. TRPC6 and TRPC^S814A^ were transfected in HEK293T cells. The cells were biotinylated with sulfo-NHS-SS-biotin, lysed, and incubated with streptavidin-agarose beads as described in Experimental Procedures. Proteins precipitated by streptavidin-agarose were separated by SDS-PAGE and detected with an anti-HA antibody (*top*). Aliquots of the cell lysates were collected before the incubation with streptavidin-agarose and analyzed directly by immunoblotting to determine the total amount of TRPC6 (*middle*) and actin (bottom) in the samples. Representative immunoblot of three independent experiments.

We next assessed the effect of the phosphorylation of Ser^814^ on the activity of TRPC6. HEK293 cells were transiently transfected with the M5 muscarinic receptor and with TRPC6 or its mutant TRPC6^S814A^. Ca^2+^ entry through TRPC6 was analyzed using the standard Ca^2+^ depletion/restoration protocol [4]. Cells were incubated for 30 s in Ca^2+^-free medium containing 0.5 mM EGTA before depleting the intracellular Ca^2+^ stores with 5 µM carbachol (CCh). Once the [Ca^2+^]_i_ had returned to the basal level (2 min after the addition of CCh), the extracellular medium was replaced with medium containing 1.8 mM CaCl_2_. Figure 3A shows that, in the absence of extracellular Ca^2+^, CCh-induced Ca^2+^ release was similar in TRPC6-, TRPC6^S814A^-, and mock-transfected cells. When 1.8 mM CaCl_2_ was added to TRPC6- and TRPC6^S814A^-transfected cells, Ca^2+^ entry raised the [Ca^2+^]_i_ to a plateau level of approximately 250 nM and 260 nM, respectively (Fig. 3A). In mock-transfected cells, Ca^2+^ entry raised the [Ca^2+^]_i_ to a lower plateau level of approximately 210 nM. Figure 3B shows the relative net Ca^2+^ entry, which was calculated by subtracting the basal [Ca^2+^]_i_ from the maximal [Ca^2+^]_i_ recorded after the extracellular Ca^2+^ had been restored. Compared to the endogenous net Ca^2+^ entry in mock-transfected cells, the expression of TRPC6 enhanced CCh-induced net Ca^2+^ entry to 127±15% (n  =  6). Expression of TRPC6^S814A^ gave similar results, with an increase of CCh-induced net Ca^2+^ entry reaching 131±17% (n  =  6). The substitution of Ser^814^ for Ala did not modify the level of expression of TRPC6 nor its amount at the plasma membrane (Fig. 4). These results suggested that the basal phosphorylation of TRPC6 on Ser^814^ does not affect channel activity.

**Figure 5:**
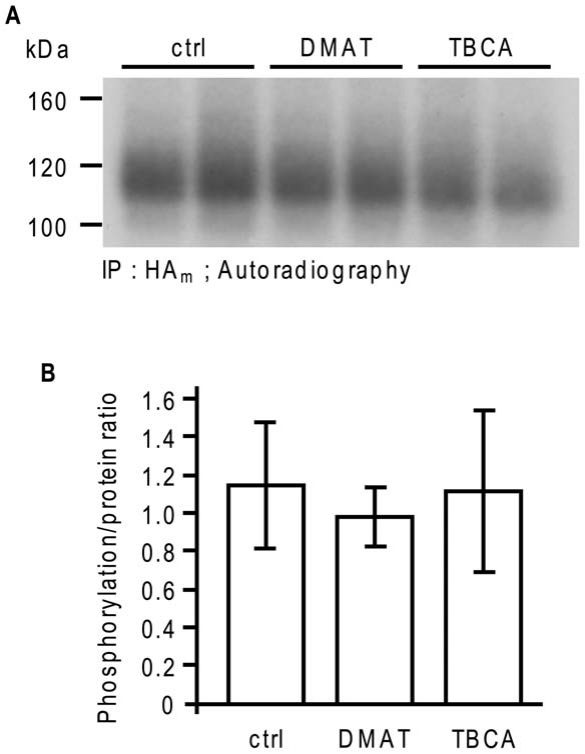
CK2 does not phosphorylate TRPC6. **A**, T6.11 cells were labeled in the presence or not of 10 µM DMAT or 10 µM TBCA for 4 h before the cells were lysed and TRPC6 was immunoprecipitated and separated by SDS-PAGE. The autoradiogram is representative of six independent experiments. **B**, The densitometric analyses of autoradiograms and Western blots (not shown) from **A** and the calculated ratios are relative to the control (100%). The histogram represents the average ± SD of six independent experiments.

An analysis of the amino acid sequence surrounding Ser^814^ of TRPC6 revealed that this region is a consensus site for CK2 substrate recognition (Ser/Thr-X-X-Glu/Asp) [24]. To verify whether CK2 is responsible for the phosphorylation of Ser^814^ of TRPC6 under basal conditions, we used two selective CK2 inhibitors, DMAT (2-dimethylamino-4,5,6,7-tetrabromo-1H-benzimidazole) and TBCA ((E)-3-(2,3,4,5-tetrabromophenyl)acrylic acid). T6.11 cells were metabolically labeled with inorganic ^32^P for 4 h in the presence of either 10 µM DMAT or 10 µM TBCA. TRPC6 was then immunoprecipitated with an anti-HA antibody and size fractionated by SDS-PAGE, and its phosphorylation state was revealed by autoradiography. Figure 5A shows that neither DMAT nor TBCA prevented or attenuated the phosphorylation state of TRPC6. The relative phosphorylation of TRPC6 incubated with DMAT and TBCA was 0.95±0.22% and 1.06±0.32%, respectively, of its phosphorylation state in untreated cells (Fig. 5B).

**Figure 6:**
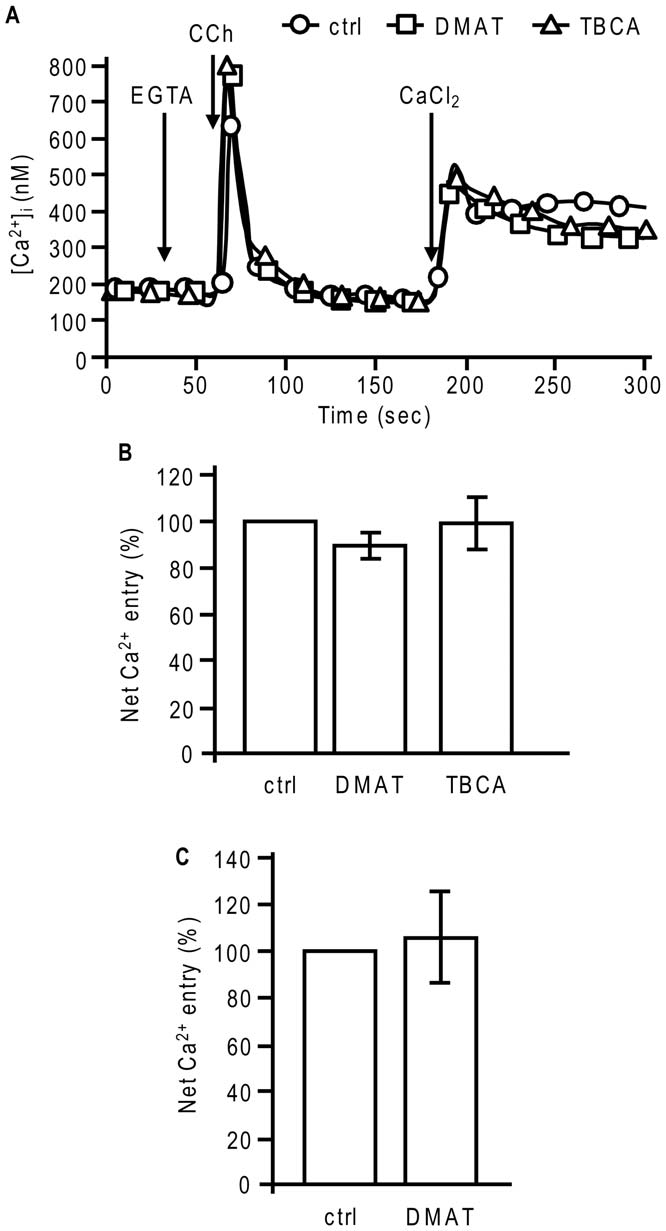
The inhibition of CK2 with specific inhibitors does not alter TRPC6 activity. **A**, Fura-2-loaded T6.11 cells were left untreated (circles) or were treated for 4 h with 10 µM DMAT (squares) or 10 µM TBCA (triangles). CCh (5 µM) induced Ca^2+^ release in the absence of extracellular Ca^2+^ while Ca^2+^ restoration 2 min later induced Ca^2+^ entry. DMAT and TBCA concentrations were maintained during the assay. The graphs are representative of 176 to 227 cells from one typical experiment. **B**, Net maximal Ca^2+^ entries from **A** were calculated as in Figure 3 and are relative to the control (100%). The histogram represents the average ± SD of four independent experiments. **C**, The same protocol was carried out on A7r5 cells using 100 nM AVP instead of CCh. The net maximal Ca^2+^ entry was calculated relative to the control (100%). The histogram is the average ± SD of three independent experiments.

Despite the fact that CK2 did not directly alter the phosphorylation state of TRPC6, we investigated whether this kinase could have a possible indirect role in Ca^2+^ signaling. In T6.11 cells, incubation for 4 h with DMAT and TBCA potentiated CCh-induced Ca^2+^ release by approximately 30%. However, after calcium restoration, DMAT and TBCA did not significantly alter CCh-induced Ca^2+^ entry (Fig. 6A). Compared to untreated cells, net CCh-induced Ca^2+^ entry in DMAT-treated cells was 89±5%, whereas it was 99±11% in TBCA-treated cells. A possible role for CK2 was also investigated in A7r5 cells. These cells endogenously express a high level of TRPC6, and their vasopressin (AVP)-induced Ca^2+^ entry is mostly through TRPC6 [25], [26], [27]. In A7r5 cells, a 4 h preincubation with 10 µM DMAT did not modify AVP-induced Ca^2+^ release (data not shown) or AVP-induced Ca^2+^ entry (Fig. 6C). These results thus exclude a role for CK2 in the direct and/or indirect regulation of TRPC6 activity.

## Discussion

Basal phosphorylation of TRPC6 has been previously observed in resting platelets and in TRPC6 stably expressed in HEK293 cells [28], [29], but the phosphorylated residue has not been identified. Using a mass spectrometry approach, we found out that Ser^814^ was phosphorylated under basal conditions in HEK293 cells. Mutation of the Ser^814^ into alanine caused a 50% decrease of basal incorporation of ^32^P in TRPC6. This phosphorylation occurred during metabolic labeling experiments, in which cells were serum starved for 4 hours and not exposed to any kind of stimulus. The 50% decrease of basal incorporation of ^32^P in TRPC6^S814A^ suggests that Ser^814^ is a major phosphorylation site under unstimulated conditions and that at least one other site is phosphorylated. Our MS/MS approach yielded 78% coverage of the intracellular regions of TRPC6. However, due to technical limitations, some large peptides were not identified. Of these, three peptides were located in the N-terminus, two peptides were located in intracellular loops, and one peptide was located in the C-terminus. Overall, these uncovered regions contained 17 phosphorylatable residues (Ser, Thr, or Tyr). Further studies are needed to verify whether one of these residues is phosphorylated under unstimulated conditions.

A recent study, which used an MS/MS approach similar to ours, revealed that *Drosophila* TRP is phosphorylated under unstimulated (dark) conditions [30]. They showed that Ser^721^ and Ser^936^ are highly phosphorylated in the dark, and that Ser^936^ is dephosphorylated in the presence of light, a phenomenon that requires the activation of the phototransduction cascade. Our study showed that Ser^814^ of TRPC6 is phosphorylated under unstimulated conditions. However, the mutation of Ser^814^ into Ala did not significantly modify the activity, the level of expression, nor the trafficking of TRPC6. Ser^814^ is localized in a highly hydrophilic region within the C-terminus of TRPC6, between the proline rich motif (amino acids 759-770), which binds FKBP12 [31], and the CIRB domain (amino acids 838-872), which binds calmodulin and inositol 1,4,5-trisphosphate receptor [32], [33], [34]. Thus, the phosphorylated Ser^814^ might play a role in the interaction of those proteins with TRPC6 or the folding and trafficking of the protein, which could be masked in recombinant cell model. To demonstrate the significance of this phosphorylation event in a non-recombinant cell model, we need to identify the kinase involved in the phosphorylation of Ser^814^. A sequence analysis of TRPC6 revealed that Ser^814^ is within a consensus site for phosphorylation by CK2 (S/T-X-X-D/E). Furthermore, CK2 favors an acidic environment as indicated by the results of Meggio *et al.*, who showed that any acidic residue in positions −2 to +7 favors phosphorylation by CK2 [24]. In TRPC6, a glutamate in position +2 and an aspartate in position +3 of Ser^814^ constitute a favorable acidic environment for CK2. CK2 is also known to exhibit constitutive activity, making it a potential kinase for the basal phosphorylation of Ser^814^. Moreover, the subcellular location and functioning of TRPP2 is modulated by the phosphorylation by CK2 [35], [36]. CK2 can phosphorylate the NR2B subunit of NMDAR, decreasing its surface expression [37], or NHE3, stimulating its basal activity [38]. However, we showed that the inhibition of CK2 by DMAT and TBCA does not reduce the basal phosphorylation level of TRPC6 or TRPC6^S814A^. We also verified whether CK2 could indirectly regulate Ca^2+^ signaling in TRPC6-expressing cells. It has previously been shown that the calcium-sensitive potassium channel K_Ca_2.2 loses Ca^2+^ sensitivity following calmodulin phosphorylation by CK2 [39]. However, we showed that inhibition of CK2 does not modify CCh-induced Ca^2+^ entry into HEK293 cells stably expressing TRPC6 (T6.11) despite the fact that it does enhance CCh-induced Ca^2+^ release. Similarly, in A7r5 cells expressing high levels of TRPC6, the inhibition of CK2 with DMAT did not affect AVP-induced Ca^2+^ release or Ca^2+^ entry. We thus showed that CK2 does not modulate the activity of TRPC6.

In summary, we showed that the serine in position 814 of TRPC6 is phosphorylated under basal conditions in HEK293 cells, and that it accounts for one of two distinct basal phosphorylation sites. Our results further showed that CK2 is not the kinase responsible for these phosphorylations, and that CK2 does not directly or indirectly modify TRPC6 activity. Further studies are needed to demonstrate the significance of the phosphorylation of Ser^814^ under unstimulated conditions and to identify the kinase responsible for this phosphorylation.

## Materials and Methods

### Material

Cell culture media, serum, Hepes, trypsin, Opti-MEM I, LipofectAMINE 2000, and Zero Blunt Topo PCR cloning kits were purchased from Invitrogen (Burlington, ON, Canada). Phosphate-free culture media and G418 were from Wisent (St-Bruno, QC, Canada). NP-40 was from Roche (Laval, QC, Canada). HEK293 and A7r5 cell lines were from ATCC (Manassas, VA, USA). DMAT, TBCA, CCh, AVP, phosphatase cocktail inhibitor set #2, and fura 2/AM were from Calbiochem (San Diego, CA, USA). Rabbit polyclonal and mouse monoclonal anti-HA-specific antibodies were from Covance (Berkeley, CA, USA). Peroxidase-conjugated donkey anti-rabbit antibodies, peroxidase-conjugated sheep anti-mouse antibodies, protein A-sepharose CL-4B, and Biomax MR films were from GE Healthcare (Baie d'Urfé, QC, Canada). Western Lightning Chemiluminescence Reagent Plus, 0.2 µm nitrocellulose membranes, and ^32^P-orthophosphoric acid were from Perkin–Elmer Life Sciences (Woodbridge, ON, Canada). All primers and oligonucleotides were from Integrated DNA Technologies (Coralville, IA, USA). Phusion High-Fidelity DNA polymerase was from Finnzymes (Espoo, Finland). Restriction enzymes, PNGase F, and T4 DNA ligase were from New England Biolabs (Pickering, ON, Canada). Unless otherwise stated, all other reagents were from Sigma (Oakville, ON, Canada) or Laboratoire MAT (Quebec City, QC, Canada).

### Mass spectrometry

#### Protein in-gel digestion

Excised acrylamide bands containing immunoprecipitated and separated TRPC6 were sent to the Proteomics Platform of the Eastern Quebec Genomics Center (CHUL Research Center, Quebec City, QC, Canada) for MS/MS analysis. Proteins were extracted from gels, placed in 96-well plates, and washed with water. Tryptic digestion was performed using a MassPrep liquid handling robot (Waters, Milford, USA) according to the manufacturer's specifications and the protocol of Shevchenko *et al.* [14] with the modifications suggested by Havlis *et al.* [15]. Briefly, proteins were reduced with 10 mM DTT and alkylated with 55 mM iodoacetamide. The trypsin digestion was performed using 105 mM modified porcine trypsin (sequencing grade, Promega, Madison, WI, USA) at 58°C for 1 h. Digestion products were extracted using 1% formic acid and 2% acetonitrile followed by 1% formic acid and 50% acetonitrile. The recovered extracts were pooled, vacuum centrifuge dried, resuspended in 8 µl of 0.1% formic acid, and 2 µl was analyzed by mass spectrometry.

#### Mass spectrometry

Peptide samples were separated by online reversed-phase nanoscale capillary liquid chromatography and analyzed by electrospray mass spectrometry. The experiments were performed using a Thermo Surveyor MS pump connected to a LTQ linear ion trap mass spectrometer (ThermoFisher, San Jose, CA, USA) equipped with a nanoelectrospray ion source (ThermoFisher). The peptides were separated on a PicoFrit column (BioBasic C18, 10 cm×0.075 mm internal diameter, New Objective, Woburn, MA, USA) with a 2-50% linear gradient of solvent B (acetonitrile, 0.1% formic acid) for 30 min at a flow rate of 200 nL/min (obtained by flow-splitting). Mass spectra were acquired using a data dependent acquisition mode using Xcalibur software version 2.0. Each full scan mass spectrum (400 to 2000 m/z) was followed by collision-induced dissociation of the seven most intense ions. The dynamic exclusion (30 s exclusion duration) function was enabled, and the relative collisional fragmentation energy was set at 35%. All MS/MS samples were analyzed using Mascot (Matrix Science, London, UK; version 2.2.0) set up to search for the sequence of mouse TRPC6 assuming trypsin as the digestion enzyme. Mascot was searched with a fragment ion mass tolerance of 0.50 Da and a parent ion tolerance of 2.0 Da. The iodoacetamide derivative of cysteine was specified as a fixed modification, and the oxidation of methionine and the phosphorylation of serine, threonine, and tyrosine were specified as variable modifications. Four missed cleavages were allowed.

#### Criteria for protein identification

Scaffold (version 2.00.05, Proteome Software Inc., Portland, OR, USA) was used to validate MS/MS-based peptide and protein identifications. Peptide identifications were accepted if they exceeded 95% probability as specified by the Peptide Prophet algorithm [16]. Protein identifications were accepted if they exceed 95% probability and contained at least two identified peptides. Protein probabilities were assigned by the Protein Prophet algorithm [17]. Proteins that contained similar peptides and could not be differentiated based on the MS/MS analysis alone were grouped to satisfy the principles of parsimony. For phosphopeptides, a pronounced neutral loss of phosphoric acid from the precursor ion and/or fragment ions was required as well as extensive coverage of the b and y series. In addition, the phosphopeptide spectra were submitted to Ascore to identify the phosphorylation site(s) on the peptide. Ascore measures the probability of correct phosphorylation site localization based on the presence and intensity of site-determining ions in MS/MS spectra [18].

### Molecular biology

Standard molecular biology techniques were used to isolate, analyze, and clone DNA [19], [20]. Point mutations in mouse TRPC6 were introduced using a PCR-based site-directed mutagenesis approach. The PCR fragments were subcloned and amplified in the pCR-BluntII-TOPO vector using a Zero Blunt TOPO PCR cloning kit. The fragments were sequenced and reinserted into HA-tagged TRPC6 in pcDNA3.1 using the appropriate restriction enzymes and T4 DNA ligase. All constructs were confirmed by sequencing from double-stranded DNA templates using the dideoxynucleotide termination method [21].

### Cell culture and transfection

HEK293T cells and A7r5 vascular myocytes were maintained at subconfluence in DMEM supplemented with 10% fetal bovine serum, 50 U/ml of penicillin, and 50 µg/ml of streptomycin at 37°C in a humidified atmosphere containing 5% CO_2_. T6.11 cells (HEK293 stably transfected with mouse TRPC6) [4] were cultured in the same medium supplemented with 400 µg/ml G418. For transient transfections, six-well plates were treated with 0.1 mg/ml poly-L-lysine for 30 min, rinsed with PBS (137 mM NaCl, 3.5 mM KCl, 10 mM sodium phosphate buffer, pH 7.4), and air-dried. Plasmid DNA (1 µg) in 250 µL Opti-MEM I was placed in each well, to which 2.5 µL of LipofectAMINE 2000 diluted in 250 µl of Opti-MEM I was added and thoroughly mixed. After a 20 min incubation, 8×10^5^ HEK293T cells diluted in 1.5 ml of culture medium without antibiotics were added to each well. The cells were incubated for 16 h at 37°C in a humidified atmosphere containing 5% CO_2_. Twenty-four hours after the transfection, the cells from one well were transferred into a 60-mm Petri dish for metabolic labeling assays or deposited on three poly-L-lysine-treated coverslips for [Ca^2+^]_i_ measurements.

### Metabolic labeling

Stably or transiently transfected cells grown in 60 mm Petri dishes were washed once with phosphate-free DMEM and incubated for 4 h in phosphate-free DMEM supplemented with 250 µCi/ml of ^32^P-inorganic phosphate. The cells were then washed twice on ice with ice-cold PBS-EDTA (137 mM NaCl, 3.5 mM KCl, 1 mM EDTA, 17.4 mM Na_2_HPO_4_, 3.5 mM NaH_2_PO_4_) prior to being lysed.

### Immunoprecipitation and protein separation

The cells were lysed with 1 ml of ice-cold lysis buffer (1.25% NP-40, 1.25% sodium deoxycholate, 2 mM EDTA, 12.5 mM sodium phosphate, pH 7.2, 1 µg/ml of soybean trypsin inhibitor, 5 µg/ml of leupeptin, 100 µM phenylmethylsulfonyl fluoride) supplemented with a phosphatase inhibitor cocktail for 30 min on ice with gentle agitation. They were then scraped from the surface of the Petri dish and centrifuged at 15 000×g for 15 min at 4°C. The supernatant was collected and immunoprecipitated with 50 µl of protein A-sepharose beads (50% slurry) and anti-HA rabbit antibody (1∶1000) for 2 h at 4°C. The samples were then centrifuged for 1 min at 4°C at 800×g and washed twice with 500 µl of ice-cold lysis buffer. Immunoprecipitated proteins were dissolved in 40 µl of 2X Laemmli buffer and boiled for 5 min before being separated on 8% SDS-polyacrylamide gels. The gels were then either stained, dried, and exposed to a film for autoradiography, or the protein bands were transferred onto a 0.2 µm nitrocellulose membrane (400 mA for 2 h or 100 mA overnight in 150 mM glycine, 20 mM Tris-base, 20% methanol) for immunoblotting. For MS/MS analyses, stained bands corresponding to TRPC6 were excised.

### Immunoblots

The nitrocellulose membranes to which the whole cell lysates and immunoprecipitated proteins had been transferred were stained with Ponceau S (0.1% in 5% acetic acid) to visualize the marker proteins, destained in TBST (20 mM Tris-HCl, pH 7.5, 137 mM NaCl, 0.1% Tween 20) and blocked in TBST containing 5% (w/v) nonfat dry milk for either 1 h at room temperature or overnight at 4°C. The membranes were then washed and incubated in TBST for 2.5 h at room temperature or overnight at 4°C with specific primary antibodies (mouse anti-HA 1∶1000). After three washes with TBST, the membranes were incubated for 1.5 h at room temperature with TBST containing 1∶10 000 peroxidase-conjugated sheep anti-mouse IgG. The blots were washed three times with TBST, and the immune complexes were detected with a Western Lightning Chemiluminescence Reagent Plus kit using the manufacturer's protocol.

### Measurement of [Ca^2+^]_i_

We used the method described by Zhu *et al*. [22] to measure [Ca^2+^]_i_. Briefly, T6.11 [4], A7r5 (ATCC), or transfected HEK293T (ATCC) cells grown on poly-L-lysine-treated coverslips were washed twice with HBSS (120 mM NaCl, 5.3 mM KCl, 0.8 mM MgSO_4_, 10 mM glucose, 1.8 mM CaCl_2_, 20 mM Hepes, pH 7.4) and loaded with fura 2/AM (1.5 µM in HBSS) for 20 min at room temperature in the dark. After washing and de-esterifying in fresh HBSS for 20 min at room temperature, the coverslips were inserted in a circular open-bottom chamber and placed on the stage of a Zeiss Axovert microscope fitted with an Attofluor Digital Imaging and Photometry System (Attofluor Inc., Rockville, MD, USA). Isolated fura 2-loaded cells were selected and their [Ca^2+^]_i_ were measured by fluorescence videomicroscopy at room temperature using alternating excitation wavelengths of 334 and 380 nm (10 nm bandpass filters). Emitted fluorescence was monitored through a 510 nm dichroic mirror and a 520 nm long pass filter set. Free [Ca^2+^]_i_ was calculated from the 334/380 fluorescence ratio using the method of Grynkiewicz *et al*. [23]. Reagents were diluted to their final concentrations in HBSS and applied to the cells by surface perfusion. Ca^2+^-free HBSS was supplemented with 0.5 mM EGTA to chelate any remaining extracellular Ca^2+^. For the transient transfections, cells were co-transfected with cDNA encoding the M5 muscarinic receptor, and only those responding to CCh were analyzed. [Ca^2+^]_i_ values were recorded every 3 s.
